# Valorization of Steel Slag and Fly Ash in Mortar: Modeling Age-Dependent Strength with Response Surface Methodology

**DOI:** 10.3390/ma18102203

**Published:** 2025-05-10

**Authors:** Xiaofeng Li, Chia-Min Ho, Huawei Li, Huaming Guo, Deliang Wang, Dan Zhao, Kun Zhang

**Affiliations:** 1 China Hebei Construction and Geotechnical Investigation Group Limited, Shijiazhuang 050227, China; mr.leexiaofeng2018@gmail.com (X.L.); wdlrain@163.com (D.W.); hbjkzd@163.com (D.Z.); 2 Key Laboratory of Groundwater Conservation of MWR, School of Water Resources and Environment, China University of Geosciences (Beijing), Beijing 100083, China; hmguo@cugb.edu.cn; 3 School of Materials Science and Engineering, Beihang University, Beijing 100191, China; hochiaminn@gmail.com; 4 State Key Laboratory of Biogeology and Environmental Geology, China University of Geosciences (Beijing), Beijing 100083, China; 5 Earth Mechanics Institute, Colorado School of Mines, Golden, CO 80401, USA; kunzhang@mines.edu

**Keywords:** steel slag, fly ash, RSM, gradient analysis, ternary blended cement

## Abstract

This study evaluates the effects of steel slag powder (SSP), fly ash (FA), and steel slag sand (SSS) on mortar compressive strength. A response surface methodology (RSM) based on central composite design (CCD) was employed to model 7-day, 28-day, and 91-day strength development, considering three quantitative variables: SSP, FA, and SSS. Statistical results confirmed the reduced cubic models were significant and predictive (R^2^ > 0.97), with non-significant lack of fit and adequate precision. Experimental results revealed that SSP and FA negatively affected early-age strength due to dilution effects and low initial reactivity, whereas SSS slightly improved it by enhancing particle packing. At later ages, SSP exhibited nonlinear effects, where moderate dosages enhanced strength, while excessive replacement led to strength reduction. SSS showed a continuously positive contribution across all ages, particularly at 91 days. Perturbation plots, contour maps, and gradient analyses indicated that SSS played a dominant role at later stages and that maintaining a proper balance among supplementary cementitious materials (SCMs) and aggregate replacements is crucial. The developed models and response surfaces provide practical guidance for designing slag-based mortars with improved mechanical properties and enhanced sustainability.

## 1. Introduction

Cement, as an indispensable binder in the traditional construction industry, has played a vital role in promoting infrastructure development and urbanization. However, it has also brought about serious environmental challenges. In 2024, global cement production reached approximately 4 billion tons [1]. Large-scale cement production, as a resource- and energy-intensive process, contributes substantially to the intensification of the greenhouse effect through significant carbon dioxide (CO_2_) emissions arising from multiple sources [2], including process emissions from the calcination of limestone, combustion-related emissions due to fossil fuel use in high-temperature operations, emissions from electricity consumption, and indirect emissions associated with the transportation and processing of raw materials [3]. It is estimated that the production of one ton of cement emits approximately 0.9 tons of CO_2_ [4]. In 2023, global carbon emissions totaled 36.8 billion tons, with the cement industry accounting for approximately 7–8% of this total [5]. Therefore, reducing cement consumption and developing low-carbon alternative materials have become critical pathways for the building materials sector to achieve carbon peaking and carbon neutrality goals [6].

Supplementary cementitious materials (SCMs) are widely used to partially replace cement in concrete production, which not only reduces cement demand and associated carbon emissions but also improves the mechanical and durability properties of cement-based materials [7,8,9]. Most SCMs are derived from industrial by-products or solid waste, such as fly ash (FA), SSP, and ground granulated blast furnace slag (GGBFS), and they typically possess either cementitious or pozzolanic activity [10,11,12]. The effects of different SCMs on concrete performance vary depending on their chemical composition and physical morphology [13]. Their mechanisms of action include the filler effect (pore filling and densification) [14], pozzolanic reaction (reaction with Ca(OH)_2_ to form C-S-H) [15], latent hydraulic activity (hydration under activation) [16], early hydration promotion (acceleration of cement hydration), pH regulation (alkaline environment maintenance) [17], ettringite formation through sulfate reaction (structure densification) [18], and carbonation (pore refinement and durability enhancement) [19]. Rational selection and synergistic blending of SCMs are crucial for developing high-performance and low-carbon concrete.

Steel slag, a solid by-product generated during steelmaking, is considered a promising cement substitute due to its latent hydraulic properties and its rich content of CaO and silicate phases [20]. Extensive research has shown that steel slag, when used as a single SCM, can contribute positively to strength development in cementitious materials [21,22]. However, the main hydraulic phase in steel slag, γ-C_2_S, is thermodynamically stable and exhibits much lower reactivity than β-C_2_S in Portland cement, resulting in slow hydration and limited early-age strength contribution [23]. Therefore, physical or chemical activation is often necessary to enhance its cementitious performance [24,25,26]. Despite its low early reactivity, the slow and continuous hydration of steel slag can facilitate sustained C-S-H gel formation, leading to improved pore structure, increased matrix densification, and steady long-term strength development. In addition, sulfate activation can promote the formation of more ettringite, and CO_2_ mineralization can further enhance the compactness of the matrix. To maximize the contribution of steel slag to strength development and improve the utilization efficiency of industrial solid waste, it is often used in combination with other SCMs such as FA, GGBFS, red mud (RM), and waste glass powder (WGP) to partially replace cement [27,28,29,30]. The synergistic mechanisms and enhancement effects of different material combinations vary, mainly in terms of hydration reaction kinetics, pore structure optimization, and long-term mechanical performance. For instance, in systems combining steel slag (SS) with GGBFS, the high-alkaline environment provided by SS promotes the activation of glassy SiO_2_ and Al_2_O_3_ in slag, thereby accelerating early hydration and generating large amounts of C-S-H gel [31]. In SS–RM systems, the presence of RM enhances the reactivity of aluminosilicate components within the matrix, leading to increased formation of both C-S-H gel and ettringite, which fill internal pores and improve the internal structure and mechanical properties of the composite [32]. In SS-WGP systems, SS can promote the early hydration of ordinary Portland cement (OPC), and at later stages, Ca(OH)_2_ released from SSP hydration can activate the pozzolanic reaction of WGP, thereby improving the overall hydration process and microstructural development of the system [33].

Fly ash, a combustion by-product from coal-fired power plants, possesses considerable pozzolanic activity and has been widely used as a mineral admixture to partially replace cement [34]. It mainly consists of amorphous, spherical particles rich in Si and Al [35]. The effect of FA on the mechanical properties of cementitious materials is dual in nature. At moderate replacement levels (typically 10–30%), early strength may slightly decrease due to delayed reactivity, but as hydration progresses, increased pozzolanic reactions generate more C-S-H gel, leading to sustained strength development. At 28 days and beyond, compressive strength often matches or exceeds that of control mixes [36]. Furthermore, FA enhances resistance to sulfate attack, mitigates alkali–silica reaction (ASR) [37], and improves chloride penetration resistance [38]. These improvements are primarily attributed to pore structure refinement and the reduction in calcium hydroxide content. In recent years, growing attention has been paid to synergistic use of FA with other industrial by-products [39]. Nevertheless, the reactivity of FA varies depending on its origin, combustion process, chemical composition, and glass phase content, which may lead to performance fluctuations [40,41].

With the depletion of natural sand and the growing environmental concerns associated with river sand mining [42], developing alternative fine aggregates from industrial by-products has become a sustainable strategy in concrete technology [43]. Coarser steel slag particles, after crushing, screening, and stabilization treatment, can serve as fine aggregates to replace natural sand. These particles typically have rough surfaces, high angularity, and strong mechanical interlock capacity [44]. Due to their high hardness, steel slag particles can form a rigid skeleton in hardened concrete, enhancing stiffness, elastic modulus, and mechanical stability. Steel slag sand (SSS) also contains latent hydraulic constituents (e.g., C_2_S and C_3_S) that may undergo delayed hydration under alkaline conditions, forming C–S–H gels that fill pores and densify the microstructure, contributing to long-term strength gain [45]. Although its reactivity is lower than traditional binders, it offers synergistic effects when combined with cement hydration, especially by improving the interfacial transition zone (ITZ) and bond strength [46].

Response surface methodology (RSM) is a statistical and mathematical approach designed to model and analyze problems in which multiple independent variables influence a response of interest [47]. RSM integrates regression modeling and graphical interpretation to evaluate the effects of variables and their interactions, and to optimize the response. Its core idea is to design a set of scientifically structured experiments that, with fewer trials, allow for the construction of a predictive model and response surface to analyze the main effects, interaction effects, and nonlinear trends [48]. In cement-based material research, RSM is particularly advantageous for evaluating synergistic effects among multiple SCMs. For instance, analyzing a single material’s effect on strength can be limited and partial [49,50], whereas RSM allows for simultaneous investigation of multiple combinations (binary or ternary), providing a comprehensive understanding of interactive mechanisms on mechanical performance.

Most previous studies have focused on the isolated replacement of either cementitious materials or aggregates, with limited attention paid to the synergistic effects of simultaneous “binder-aggregate multi-component substitution” on the strength development of mortar at different curing ages. In this study, SSP and FA were used to synergistically replace cement, while SSS was employed as a substitute for natural river sand to prepare mortar specimens. Based on a multivariable experimental design (with varying replacement ratios of solid waste materials) and compressive strength testing at 7 days, 28 days, and 91 days, an RSM-based regression model was established to describe the relationship between material replacement ratios and mechanical performance. Multi-way analysis of variance (ANOVA), response surface analysis, and gradient analysis were conducted to systematically investigate the effects and interactions of SSP, FA, and SSS replacement ratios on mortar compressive strength. The developed model was further evaluated in terms of fitting accuracy and predictive performance, confirming its reliability. On this basis, the synergistic effects of the SSP–FA composite binder system under different levels of SSS replacement were further analyzed across various curing ages. The results provide fundamental insights into the synergistic utilization of industrial solid wastes such as steel slag and FA, supporting their resource-efficient application. This approach offers significant potential to reduce cement and natural aggregate consumption, lower energy and resource use, and contribute to the achievement of carbon peak and carbon neutrality goals.

## 2. Materials and Methods

### 2.1. Materials

Ordinary Portland cement (OPC) complying with BS EN 197 [51] was employed as the primary binder and categorized as CEM I 42.5N. SSP and Class F FA were introduced as SCMs, partially substituting cement by mass. The SSP was produced by finely grinding air-cooled steel slag, with the resulting particles passing through a 200-mesh sieve (i.e., particle size < 75 μm). Prior research has demonstrated that the fineness of SSP plays a critical role in determining the mechanical properties and durability of cementitious composites. Finer SSP (<75 μm) typically possesses a greater fraction of reactive phases and enhanced hydration potential, thus representing a favorable range for chemical reactivity and modification [12,52]. The FA used in this study was sourced from a local coal-fired power plant and conformed to the standards set out in BS EN 450-1:2012 [53]. The chemical compositions of the binders were determined by X-ray fluorescence spectrometry, Malvern Panalytical Zetium (Malvern, UK). The chemical compositions of the binders are detailed in Table 1.

Well-graded natural river sand, with a fineness modulus of approximately 2.7 and a specific gravity of 2.6, served as the fine aggregate, conforming to BS EN 12620 [54]. In selected mixtures, a portion of the sand was replaced with SSS, which was obtained by crushing and screening coarse steel slag.

### 2.2. Mortar Preparation and Testing

The mortar mixes were prepared with a fixed water-to-binder (*w*/*b*) ratio of 0.5 and a binder-to-sand ratio of 1:3. In each formulation, cement was partially substituted by SSP and FA at replacement rates of 0%, 10%, and 20%, respectively. Likewise, natural river sand was replaced by SSS at the same substitution levels (0%, 10%, and 20% by mass). The detailed mix proportions are presented in Table 2.

All dry components were initially mixed for 2 min to achieve homogeneity, after which water was gradually added, and mixing continued for an additional 3 min. The fresh mortar was then placed into 50 mm × 50 mm × 50 mm cube molds in two layers with proper compaction. After 24 h of setting, the specimens were demolded and transferred to a water-curing environment maintained at 25 ± 2 °C until the specified testing ages.

To assess mechanical performance, compressive strength tests were carried out at 7 days, 28 days, and 91 days. The compressive strength of all mortar cubes is measured by a hydraulic universal testing machine, following the procedures stated in BS EN 12390-3 [55]. For each mix and curing period, three samples were tested, and the mean value was recorded as the representative compressive strength.

### 2.3. Mathematical Model Development

To investigate the interactive effects of SSP, FA, and SSS on the compressive strength of mortar, RSM was employed to develop predictive mathematical models. The experimental design and statistical analyses were conducted using Design-Expert 13 software.

**Figure 1:**
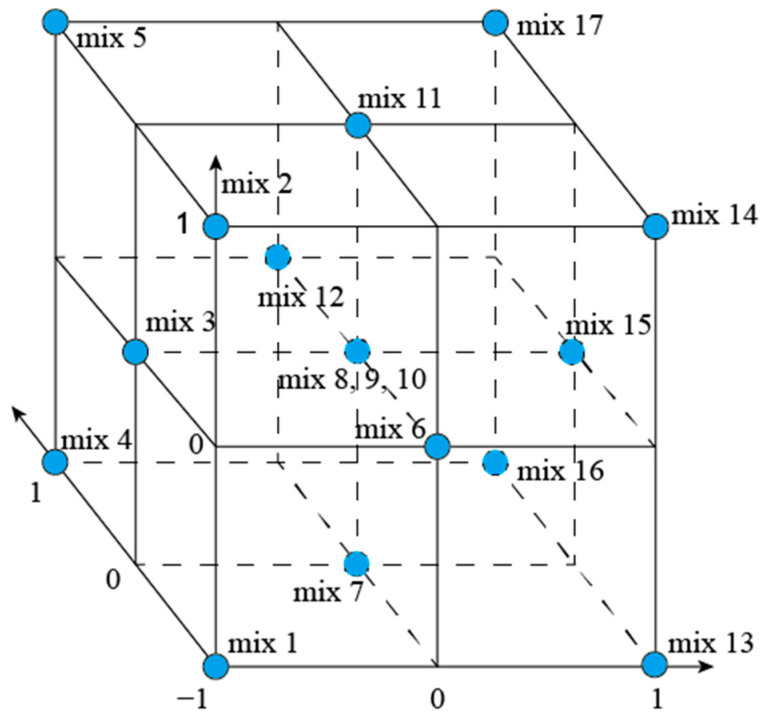
Design method of CCD.

A central composite design (CCD) was adopted due to its suitability for fitting second-order polynomial regression models with a minimal number of experimental runs. Three independent numeric factors were selected: the replacement levels of SSP and FA (as partial cement substitutes) and SSS (as a partial sand substitute). Each factor was evaluated at three levels (0%, 10%, and 20% by mass), coded as −1, 0, and +1, respectively (shown in Table 3). A face-centered CCD (α = 1) was applied to ensure all points fall within the design cube and facilitate practical interpretation of factor levels. The experimental design consisted of 14 factorial and axial (non-center) runs and 3 replicates at the center point, totaling 17 mixtures. This allowed for the estimation of both linear and quadratic effects, as well as the detection of any potential curvature in the response surface. The design method of CCD used in this study has been shown in Figure 1. The compressive strength at 7 days, 28 days, and 91 days was selected as the response variable for model development. The experimental data were fitted to cubic polynomial equations, and analysis of variance (ANOVA) was used to evaluate the significance of the models, individual factors, and their interactions. A significance level of *p* < 0.05 was adopted to determine statistical relevance. The coefficient of determination (R^2^), adjusted R^2^, and predicted R^2^ were used to examine model fitting and predictive performance. Adequate precision values were also checked to confirm that the signal-to-noise ratio was acceptable (greater than 4.0). No significant lack of fit (*p* > 0.05) was observed, indicating that the models were statistically adequate for prediction within the design space. The developed models were subsequently used to generate perturbation plots, interaction plots, and contour plots, which facilitated the visualization and interpretation of the individual and combined effects of SSP, FA, and SSS on mortar strength.

### 2.4. Gradient Vector Calculation

To further explore the sensitivity of compressive strength to compositional changes, gradient vectors were calculated based on the partial derivatives of the fitted response surface models. For a given polynomial model *f*(*x*, *y*, *z*), where *x*, *y*, and *z* represent the coded replacement levels of SSP, FA, and SSS, respectively,

$$f\left(x,\;y,\;z\right)=\beta_{0}+\sum_{i=1}^{3}\beta_{i}x_{i}+\sum_{i=1}^{3}\sum_{j=1}^{3}\beta_{ij}x_{i}x_{j}$$

The gradient vector ∇*f* quantifies the direction and rate of the steepest ascent or descent in strength. It is defined as:

$$\nabla f\left(x,\;y,\;z\right)=\left[\frac{\partial f}{\partial x},\;\frac{\partial f}{\partial y},\;\frac{\partial f}{\partial z}\right]$$

Each component of the vector corresponds to the first-order partial derivative with respect to a specific factor, incorporating linear, interaction, and quadratic terms from the regression equation.

$$\frac{\partial f}{\partial x_{i}}=\beta_{i}+\sum_{j-1}^{n}\beta_{ij}x_{j}+2\beta_{ii}x_{i}$$

These derivatives were computed analytically from the fitted models and evaluated numerically over a grid of design points spanning the experimental domain. The resulting vectors were then overlaid onto the two-dimensional contour plots to visualize the most influential directions in the design space. In this context, the inverse direction of each arrow represents the path toward strength enhancement, providing an intuitive reference for optimizing binder and aggregate substitution levels. This gradient-based analysis serves as a complementary tool to traditional contour mapping, offering a more quantitative interpretation of the response surface geometry.

## 3. Results and Discussion

### 3.1. Compressive Strength of Mortar

**Figure 2:**
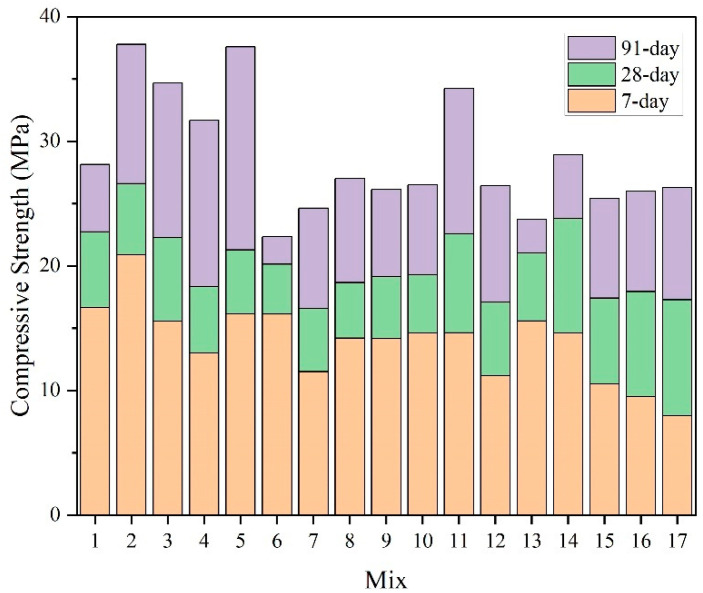
Compressive strength of eco-efficient mortar at 7 days, 28 days, and 91 days.

The development of compressive strength of eco-efficient mortar from 7 days to 91 days is illustrated in Figure 2. All strength values, including those of the three replicated mixes (Mix 6, Mix 7, and Mix 8), were obtained through laboratory testing. At 7 days, the compressive strength ranged from 7.95 to 20.87 MPa, with the lowest value recorded for Mix 17 (SSP: 20%; FA: 20%; SSS: 20%) and the highest for Mix 2 (SSP: 0%; FA: 0%; SSS: 20%). At 28 days, strength values ranged from 16.57 to 26.59 MPa, observed in Mix 7 (SSP: 10%; FA: 10%; SSS: 0%) and Mix 2, respectively. The strength gained between 7 and 28 days varied from 4.01 to 9.23 MPa. By 91 days, the strength further increased, ranging from 22.34 to 37.77 MPa, with Mix 6 (SSP: 10%; FA: 0%; SSS: 10%) and Mix 2 again representing the lowest and highest values, respectively. The increment from 28 days to 91 days ranged from 2.21 to 16.28 MPa. It is noteworthy that Mix 2 consistently achieved the highest compressive strength across all curing ages, whereas the lowest strength was associated with different mix designs at each age. Steel slag sand (SSS), with its rough surface and high hardness, improves the mechanical interlocking and forms a rigid skeleton within the concrete matrix. Additionally, its latent hydraulic activity contributes to secondary C-S-H formation, enhancing the compactness and strength of concrete. A clear difference was observed in the range of strength increments between the early (7 days to 28 days) and later (28 days to 91 days) curing periods. This disparity can be attributed to the pozzolanic reaction of fly ash, which contributes more significantly to strength development at later ages [56,57]. This trend is further supported by the mix compositions: most mixes without FA showed strength gains below 5.42 MPa from 28 days to 91 days, while all mixes incorporating FA exhibited corresponding gains exceeding 7.02 MPa.

### 3.2. RSM Analysis

#### 3.2.1. Analysis of Variance

The experimental data are shown in Table 4 and were analyzed using RSM, and reduced cubic models were developed to predict the compressive strength of mortar at 7 days, 28 days, and 91 days. ANOVA has been shown in Table 5. ANOVA demonstrates all three models were found to be highly significant (*p* < 0.0001), with large F-values of 40.86, 79.51, and 165.77 for 7 days, 28 days, and 91 days, respectively, indicating strong correlations between the selected input factors and the responses. The goodness-of-fit statistics confirmed the adequacy of the models. At 7 days, the model yielded an R^2^ of 0.9761, an adjusted R^2^ of 0.9522, and a predicted R^2^ of 0.9137. The adequate precision of 26.23 far exceeded the recommended threshold of 4. Similarly, the 28-day model showed an R^2^ of 0.9943, an adjusted R^2^ of 0.9818, and a predicted R^2^ of 0.9358, while the 91-day model demonstrated the highest statistical robustness, with an R^2^ of 0.9980, an adjusted R^2^ of 0.9920, and a predicted R^2^ of 0.9351. These results indicate that all models demonstrated excellent fitting quality and predictive reliability. Notably, the 7-day model exhibited the smallest gap between adjusted R^2^ and predicted R^2^, suggesting relatively consistent predictive performance even at early curing stages. In contrast, although the 28-day and 91-day models achieved higher R^2^ and adjusted R^2^ values, the slightly larger gaps between adjusted and predicted R^2^ imply that the predictive capability became marginally less stable at later ages. Nevertheless, the high overall R^2^ values and the acceptable predicted R^2^ values at all ages confirm the strong robustness and generalization capacity of the developed models. All models passed the lack-of-fit test (*p* > 0.1), confirming the reliability of the fitted surfaces within the experimental design space.

The regression equations in terms of coded factors are expressed as follows:

(1) $$\begin{array}{ll} f_{7-day}\left(x,y,z\right) & =13.6-2.4x-2.6y+1.57z-0.54xy-1.24xz-0.21yz+0.56y^{2}\\ & -0.96x^{2}y \end{array}$$

(2) $$\begin{array}{ll} f_{28-day}\left(x,y,z\right) & =18.77-2.42x-1.53y+2.99z-0.59xz-0.54yz+1.29x^{2}\\ & +1.01z^{2}-0.32xyz-0.89x^{2}y-1.87x^{2}z+1.31xy^{2} \end{array}$$

(3) $$\begin{array}{ll} f_{91-day}\left(x,y,z\right) & =26.72-4.64x+2.04y+4.8z-0.46xy-1.26xz-1.08yz+3.2x^{2}\\ & -2.46y^{2}+2.59z^{2}-1.67x^{2}y-2.18x^{2}z+0.86xy^{2} \end{array}$$

Analysis of the regression coefficients provides insight into the contribution and behavior of each factor. At 7 days, SSP (*x*) and FA (*y*) both exhibited negative effects on strength, while SSS (*z*) contributed positively. This suggests that the substitution of cement with SSP and FA delays early strength development [58,59,60,61], while aggregate-level substitution with SSS may enhance early packing density [62]. Notably, the interaction terms *xy* and *xz* had negative effects, and the cubic term *x*^2^*z* indicated nonlinearity at higher replacement levels. By 28 days, the positive effect of SSS (*z*) became more dominant, while SSP and FA retained moderate negative influences. Several higher-order terms such as *x*^2^, *z*^2^, *x*^2^*z*, and *xy*^2^ became statistically significant, pointing to the increasing role of nonlinearity and interaction effects. These findings suggest that moderate levels of SCMs and SSS can enhance strength, while excessive replacement may compromise performance due to incomplete hydration or workability reduction. At 91 days, the regression model revealed a more complex response surface, with strong quadratic and cubic effects. SSP showed a marked negative impact (−4.64), indicating that excessive cement replacement may hinder long-term strength. In contrast, both FA and SSS showed positive effects, likely due to pozzolanic reactions and matrix densification over time [63,64,65]. The significance of terms like *x*^2^, *y*^2^, *z*^2^, *x*^2^*z*, and *xy*^2^ reflects the synergistic and nonlinear hydration behaviors at later ages, reinforcing that long-term performance in ternary systems is governed by coupled chemical and physical mechanisms. These results collectively demonstrate that the response of mortar strength to varying combinations of SSP, FA, and SSS is highly age-dependent and nonlinear. The RSM models not only provide accurate predictive tools but also offer insights into optimizing binder and aggregate compositions for desired mechanical performance across curing stages.

#### 3.2.2. Strength Development Analysis

To visualize the influence of SSP, FA, and SSS on compressive strength development across different curing ages, two-dimensional contour plots were generated for 7 days, 28 days, and 91 days, accompanied by overlaid gradient vectors. In these plots, the arrows indicate the direction of maximum rate of decrease in strength; therefore, the inverse direction of each arrow represents the path toward higher strength, offering insight into the most favorable compositional trends.

**Figure 3:**
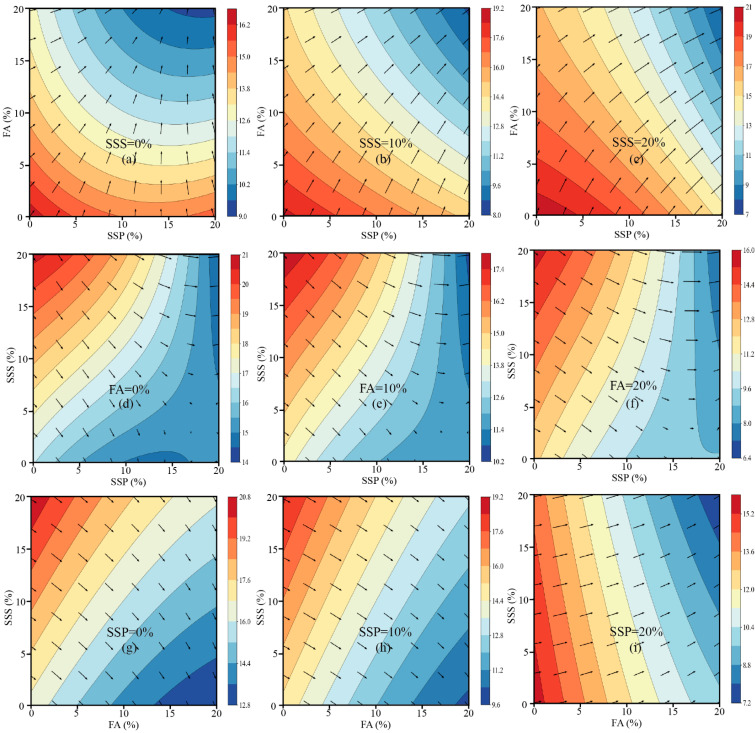
Contour plots and strength gradients of 7-day compressive strength under various combinations of SSP, FA, and SSS.

**Figure 4:**
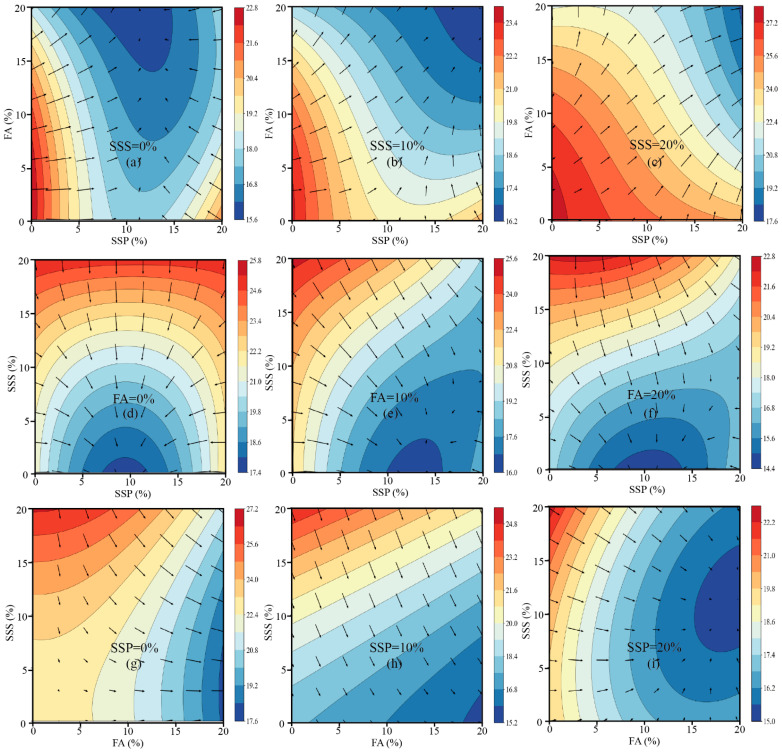
Contour plots and strength gradients of 28-day compressive strength under various combinations of SSP, FA, and SSS.

**Figure 5:**
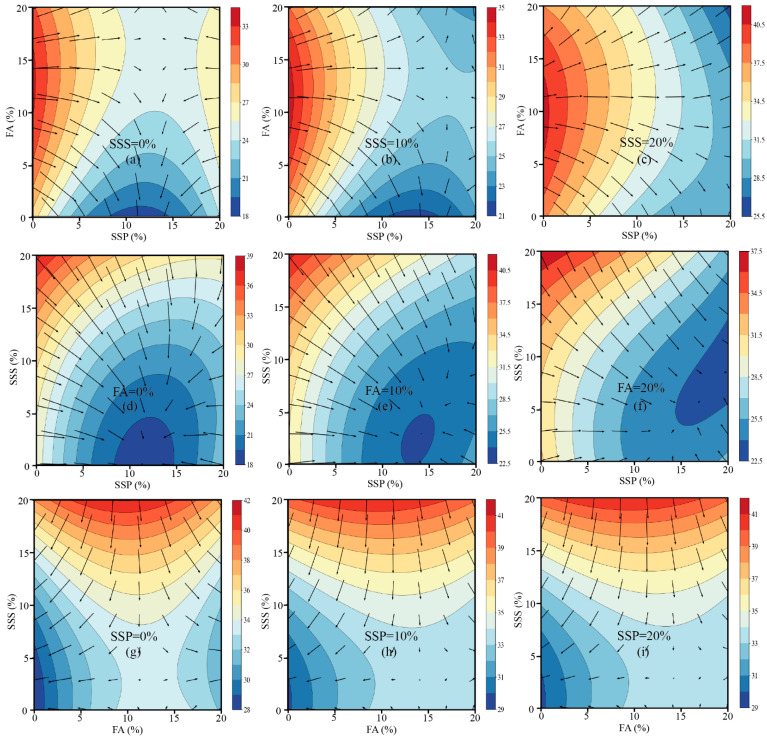
Contour plots and strength gradients of 91-day compressive strength under various combinations of SSP, FA, and SSS.

At 7 days (Figure 3), the contour plots demonstrate that the compressive strength of mortar decreases progressively with increasing contents of SSP and FA, while the positive contribution of SSS becomes evident. Most gradient vectors point diagonally from the lower-left corner (low SSP and FA contents) to the upper-right corner (high SSP and FA contents), indicating that minimizing SSP and FA replacement while maximizing SSS incorporation is favorable for early-age strength development. This trend is consistent with the negative regression coefficients associated with SSP and FA at this age, reflecting their limited early hydration reactivity and dilution effects. Moreover, the flatter gradient fields observed at higher SSS replacement levels (e.g., 20%) suggest that the system becomes less sensitive to variations in SCM content when sufficient SSS is present, likely due to enhanced particle packing and pore structure refinement. This finding highlights the significant role of aggregate-level optimization in mitigating the early strength loss associated with high SCM dosages. At 28 days (Figure 4), the response surfaces reveal a more complex behavior, characterized by the emergence of curved contour lines, localized strength ridges, and valleys. These features reflect the increased significance of nonlinear and interaction effects, as confirmed by the regression model where quadratic terms (x^2^, z^2^) and cross-interaction terms (x^2^z and xy^2^) became statistically significant [48]. The optimal strength paths identified from the gradient vectors suggest that moderate SSP replacement levels (approximately 5–10%), combined with elevated SSS contents (>10%), are most beneficial for achieving improved compressive strength at this stage. Meanwhile, FA shows a moderate negative influence, and excessive FA levels (>15%) appear to reduce the strength. This behavior can be attributed to the delayed pozzolanic reaction of FA, which only partially compensates for the dilution effect within 28 days. Overall, the plots at 28 days underline the importance of achieving a balance between binder dilution and aggregate packing to optimize mechanical performance. At 91 days (Figure 5), the contour surfaces evolve into smoother profiles, with larger high-strength plateaus and less pronounced gradient directions. The vectors suggest that increasing FA and SSS together, while maintaining SSP at a lower or moderate level, is most beneficial for long-term strength. These observations are supported by the strong positive effects of FA (+2.04) and SSS (+4.80), and the continued negative effect of SSP (−4.64) in the 91-day model. The reduced density and length of gradient vectors across this surface indicate diminished sensitivity of the system to compositional changes, reflecting hydration maturity and the formation of a stable microstructure. Collectively, the gradient-based contour analysis corroborates the statistical modeling results and offers a visual framework for interpreting how different material combinations influence strength evolution. While early-age performance is primarily governed by dilution and packing effects, mid- to late-age strength is shaped by complex synergistic interactions and the latent hydraulic activity of the supplementary cementitious materials [66]. The direction and magnitude of the gradients provide a quantitative basis for optimizing mix designs to target desired strength outcomes over time.

#### 3.2.3. Main Effect Analysis

**Figure 6:**
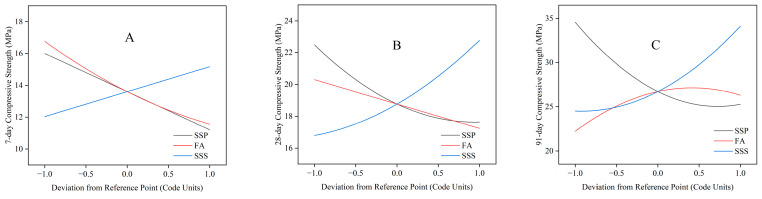
Perturbation plots showing the effects of SSP, FA, and SSS replacement levels on mortar compressive strength at (**A**) 7 days, (**B**) 28 days, and (**C**) 91 days.

The perturbation plots (Figure 6) across 7 days, 28 days, and 91 days provide an integrated perspective on the evolving influence of SSP, FA, and SSS on the compressive strength of blended mortar. At early age (Figure 6A), both SSP and FA demonstrate steep negative slopes, confirming their significant suppressive effects on strength development due to limited early-stage reactivity. FA appears to exert a slightly stronger influence, likely due to its lower initial pozzolanic activity compared to cement. In contrast, SSS shows a marginally positive effect, suggesting that its filler role contributes modestly by refining the pore structure rather than through direct chemical hydration. By 28 days (Figure 6B), a transitional behavior is observed. The negative influence of SSP persists but begins to diminish in magnitude, implying partial activation of its latent hydraulic properties. FA also continues to exert a downward effect, although the slope is more moderate, potentially reflecting the onset of pozzolanic reactions [56,67]. Notably, SSS transitions into a clearly beneficial component, with its contribution to compressive strength becoming increasingly pronounced, consistent with microstructural enhancement through improved packing density and reduced porosity. At 91 days (Figure 6C), the system exhibits a more complex and nonlinear response. SSP maintains its overall negative impact, though the curve flattens at higher dosages, indicating diminishing marginal losses. FA, on the other hand, demonstrates a weak parabolic trend with strength improvements near moderate replacement levels, highlighting the delayed but meaningful contribution of pozzolanic activity. SSS consistently delivers a robust positive influence, exhibiting a steadily rising curve, which confirms its structural enhancement capabilities and long-term stability in supporting hydration products.

Taken together, these perturbation plots underscore a temporal shift in dominant influencing factors. While SSP and FA require cautious dosage control due to their age-dependent chemical reactivity, SSS emerges as a structurally reliable component, particularly effective in long-term performance enhancement. These findings reinforce the necessity of tailored mix design strategies that leverage physical synergies among SCMs to ensure balanced strength development over time.

#### 3.2.4. Interaction Analysis Among SSP, FA, and SSS

The perturbation plots for 7-day, 28-day, and 91-day compressive strength provide a clear visualization of the relative influence of each individual factor, which are SSP, FA, and SSS, on the mortar strength response, with all other variables held constant at the reference point.

**Figure 7:**
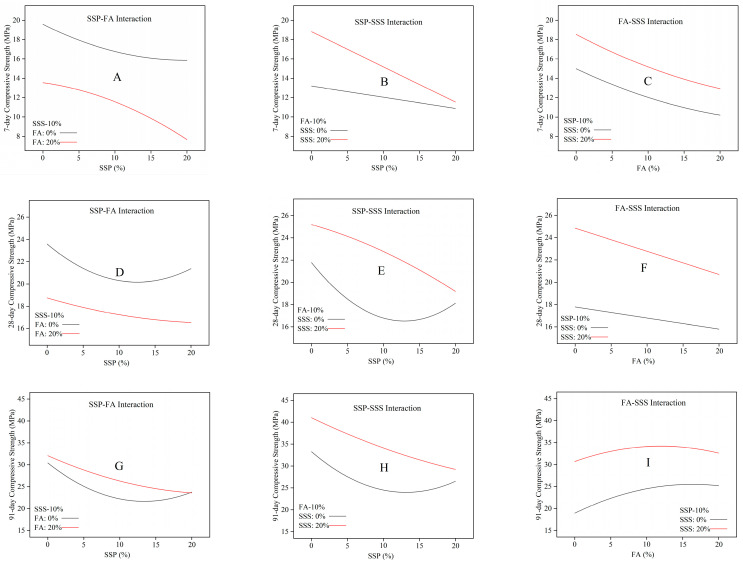
Interaction plots illustrating the combined effects of SSP, FA, and SSS on mortar compressive strength at (**A**–**C**) 7 days, (**D**–**F**) 28 days, and (**G**–**I**) 91 days.

A holistic examination of the interaction plots (Figure 7A–I) reveals the complex and age-dependent interplay among SSP, FA, and SSS in shaping mortar compressive strength. At early age (Figure 7A–C), the concurrent increase in SSP and FA induces a notable decline in strength, particularly when both are applied at high replacement ratios. This compounded dilution effect highlights the vulnerability of the hydration system when multiple low-reactivity binders are used simultaneously. SSS, while beneficial in improving matrix packing, shows limited capacity to counterbalance the reactivity shortfall at this stage. By 28 days, the interaction effects become more nuanced. As evidenced in Figure 7D–F, SSS begins to exhibit a more pronounced structural contribution, particularly in systems with moderate FA content. The strength response to SSP shifts from linear to nonlinear under varying FA levels, suggesting that SSS enables better utilization of SSP’s latent reactivity within a more stable microstructure. However, when the FA content is elevated, this synergy weakens, revealing a threshold beyond which neither SSS nor SSP can independently sustain performance without sufficient cementitious activity. At 91 days, the long-term behavior of these interactions becomes evident. Figure 7G–I demonstrate that the pozzolanic activation of FA and delayed reactivity of SSP progressively reinforce strength development, particularly when supported by the structural integration offered by SSS. The presence of SSS consistently enhances performance across FA and SSP gradients, though its impact diminishes when the reactive matrix is overly diluted. Optimal strength is observed under balanced combinations—moderate SSP and FA levels complemented by sufficient SSS—indicating that the effectiveness of each component is contingent upon the reactive–structural equilibrium of the ternary system.

Collectively, the interaction plots confirm that SSP, FA, and SSS do not function independently; rather, their combined influence is highly dependent on curing age and their relative dosages. SSS plays a crucial stabilizing role, especially at later stages, by reinforcing the system’s structural integrity. However, its positive effects are conditional upon the presence of adequate reactive phases. The results underline the importance of synergistic mix design, where optimization of both hydration potential and structural support is essential to fully exploit the performance benefits of these industrial by-products.

## 4. Recommendations

Based on the interaction analysis among SSP, FA, and SSS across different curing ages, several practical recommendations can be proposed. For early-age applications, it is advisable to limit the combined replacement level of SSP and FA, especially when rapid strength development is critical. The use of SSS as a partial sand replacement is recommended even in early stages, as it contributes to structural integrity without adversely affecting hydration kinetics. For long-term performance, a balanced ternary blend incorporating moderate levels of SSP (10–15%), FA (8–12%), and SSS (15–20%) is suggested to ensure both chemical reactivity and microstructural stability. Future studies should explore the threshold levels of binder dilution under varying curing conditions and consider the incorporation of chemical activators to enhance early reactivity of SSP–FA systems. Additionally, microstructural characterization techniques such as MIP and SEM could provide further insight into the packing efficiency and ITZ development contributed by SSS, supporting optimized design of sustainable cementitious composites.

## 5. Conclusions

This study explored the combined use of steel slag powder (SSP), fly ash (FA), and steel slag sand (SSS) as partial replacements for cement and fine aggregate in mortar, aiming to enhance mechanical performance while promoting sustainable material utilization. A central composite design (CCD) within a response surface methodology (RSM) framework was employed to model compressive strength development at 7, 28, and 91 days. Through a combination of regression modeling, analysis of variance, response surface visualization, gradient vector mapping, perturbation analysis, and single-factor effect evaluation, the mechanisms underlying the influence of each variable were systematically investigated. The key findings are summarized as follows:

(1) SSP and FA, when used as partial cement replacements, significantly reduce early-age (7-day) compressive strength due to their low initial reactivity and dilution effect.

(2) SSS, as a partial replacement for fine aggregate, consistently improves strength at all curing ages, with the most pronounced effect observed at 91 days, attributed to enhanced particle packing and filler effects.

(3) At later ages (28 days and 91 days), SSP exhibits nonlinear behavior. Moderate SSP dosages (~5–10%) contribute positively to strength, whereas excessive replacement (>15%) leads to strength reduction.

(4) Statistical modeling using reduced cubic regression models demonstrated high accuracy (R^2^ > 0.97) and reliability, with non-significant lack of fit and acceptable residual behavior across all models.

(5) Response surface, perturbation, and gradient analyses revealed optimal strength development occurs through a balanced combination of SCMs, particularly favoring high SSS and moderate SSP contents.

(6) The study provides practical guidance for designing sustainable mortar mixtures incorporating steel slag materials, offering a viable approach for reducing cement consumption while maintaining or enhancing mechanical performance.

## Figures and Tables

**Table 1:** Chemical compositions of binders.

Binder	CaO	SiO_2_	Al_2_O_3_	Fe_2_O_3_	MgO	SO_3_	K_2_O	Na_2_O
	%	%	%	%	%	%	%	%
Error	±0.1%	±0.1%	±0.1%	±0.1%	±0.2%	±0.2%	±0.2%	±0.2%
OPC	63.37	19.26	3.64	4.22	1.49	1.71	0.25	0.18
SSP	39.12	13.34	8.41	24.31	3.53	1.22	0.46	0.27
FA	4.7	48.5	25.4	7.8	2.2	0.5	3.4	1.1

**Table 2:** Mix design.

Mix	SSP%	FA%	SSS%
1	0	0	0
2	0	0	20
3	0	10	10
4	0	20	0
5	0	20	20
6	10	0	10
7	10	10	0
8	10	10	10
9	10	10	10
10	10	10	10
11	10	10	20
12	10	20	10
13	20	0	0
14	20	0	20
15	20	10	10
16	20	20	0
17	20	20	20

**Table 3:** Coding and real levels for the RSM custom model.

Variables	Symbol	Unit	Coded Factor Levels		
			−1	0	1
SSP	**x**	%	0	10	20
FA	y	%	0	10	20
SSS	z	%	0	10	20

**Table 4:** Experimental data used for establishing the RSM model of 7-day, 28-day, and 91-day compressive strength, and the predicted value calculated by the corresponding RSM model.

Mix	SSP%	FA%	SSS%	7 Days		28 Days		91 Days	
				Experimental	Predicted	Experimental	Predicted	Experimental	Predicted
1	0	0	0	16.64	16.56	22.73	22.67	28.15	28.04
2	0	0	20	20.87	20.68	26.59	26.53	37.77	37.96
3	0	10	10	15.56	16	22.26	22.48	34.68	34.56
4	0	20	0	13.00	12.86	18.34	18.27	31.7	31.86
5	0	20	20	16.14	16.14	21.29	21.25	37.57	37.46
6	10	0	10	16.12	16.76	20.13	20.3	22.34	22.22
7	10	10	0	11.50	12.03	16.57	16.79	24.63	24.51
8	10	10	10	14.21	13.6	18.65	18.77	27.02	26.72
9	10	10	10	14.15	13.6	19.13	18.77	26.15	26.72
10	10	10	10	14.62	13.6	19.3	18.77	26.51	26.72
11	10	10	20	14.63	15.17	22.55	22.77	34.23	34.11
12	10	20	10	11.18	11.56	17.07	17.24	26.43	26.3
13	20	0	0	15.58	15.32	21.04	20.99	23.73	23.92
14	20	0	20	14.60	14.48	23.82	23.77	28.9	28.8
15	20	10	10	10.53	11.2	17.41	17.64	25.4	25.28
16	20	20	0	9.52	9.46	17.93	17.87	26.01	25.9
17	20	20	20	7.95	7.78	17.27	17.21	26.28	26.46

**Table 5:** Analysis of variance (ANOVA) of RSM models.

	7 Days				28 Days				91 Days			
	Ss	Df	F-Value	*p*-Value	Ss	Df	F-Value	*p*-Value	Ss	Df	F-Value	*p*-Value
Model	149.55	8	40.86	<0.0001	123.51	11	79.51	<0.0001	360.87	12	165.77	<0.0001
x	57.74	1	126.22	<0.0001	11.76	1	83.28	0.0003	43.06	1	237.36	0.0001
y	67.70	1	147.99	<0.0001	4.68	1	33.15	0.0022	8.36	1	46.11	0.0025
z	4.90	1	10.71	0.0113	17.88	1	126.61	<0.0001	46.08	1	254.01	<0.0001
xy	2.35	1	5.15	0.053	2.75	1	19.47	0.0069	1.71	1	9.43	0.0372
xz	12.30	1	26.89	0.0008					12.60	1	69.46	0.0011
yz	0.35	1	0.77	0.4055	2.37	1	16.75	0.0094	9.37	1	51.68	0.002
x^2^					5.03	1	35.65	0.0019	27.37	1	150.86	0.0003
y^2^	1.29	1	2.82	0.1315					16.20	1	89.30	0.0007
z^2^					3.12	1	22.06	0.0054	17.92	1	98.77	0.0006
xyz					0.80	1	5.67	0.0631				
x^2^y					1.26	1	8.95	0.0304	4.46	1	24.60	0.0077
x^2^z	1.48	1	3.24	0.1096	5.62	1	39.78	0.0015	7.62	1	42.01	0.0029
xy^2^					2.76	1	19.55	0.0069	1.18	1	6.49	0.0635
Residual	3.66	8			0.7061	5			0.7256	4		
Lack of Fit	3.53	6	8.99	0.1035	0.4788	3	1.40	0.4416	0.3434	2	0.90	0.5267
Pure Error	0.1309	2			0.2273	2			0.3822	2		
Cor Total	153.21	16			124.21	16			361.59	16		
R^2^	0.9761				0.9943				0.9980			
Adjusted R^2^	0.9522				0.9818				0.9920			
Predicted R^2^	0.9137				0.9358				0.9351			
Adeq pre	26.2324				30.8467				42.2253			
MSE	0.2153				0.0416				0.0426			
RMSE	0.4640				0.2039				0.2064			
MAE	0.3765				0.1582				0.1741			

## Data Availability

The original contributions presented in this study are included in the article. Further inquiries can be directed to the corresponding author.
